# The potential role of vitamin E in patients with glucose-6-phosphate dehydrogenase deficiency: A systematic review and meta-analysis

**DOI:** 10.1097/MD.0000000000032937

**Published:** 2023-02-10

**Authors:** Omar Ahmed Abdelwahab, Khaled Akil, Ali Seif, Mahmoud Allam, Mohamed El-Sebaey Sherif, Mohamed N. Al-Alfy

**Affiliations:** a Medical Research Group of Egypt, Cairo, Egypt; b Faculty of Medicine, Al-Azhar University, Cairo, Egypt; c Faculty of Medicine, University of Aleppo, Aleppo, Syria; d Internal Medicine Department, Faculty of Medicine, Al-Azhar University, Cairo, Egypt.

**Keywords:** 6, glucose, hemolytic anemia, phosphate dehydrogenase deficiency, vitamin E

## Abstract

As an antioxidant, vitamin E (VitE) may benefit the erythrocytes by protecting glutathione from oxidation by free radicals and peroxide-generating processes.

**Methods:**

We followed the Preferred Reporting Items for Systematic Reviews and Meta-Analyses statement guidelines when reporting this systematic review. We searched 6 electronic databases (PubMed, Scopus, Web of Science, and Cochrane Library) until May 8, 2022. We included all relevant studies. According to the study design, the Cochrane assessment tool (Risk of Bias 2), Risk Of Bias In Non-randomized Studies - of Interventions checklists, and National Institutes of Health tools were used to assess the risk of bias.

Continuous data were pooled as a mean difference (MD) with a relative 95% confidence interval. The protocol was registered on PROSPERO (CRD42022333848).

**Results:**

Six studies were included in the meta-analysis with a total of 181 patients. Compared with the control group, VitE significantly improved the hemoglobin level for chronic hemolysis (MD = 2.72 g/dL, *P* < .0001) and for acute hemolysis (MD = 1.18 g/dL, *P* < .0001). It also decreased the reticulocyte level for chronic hemolysis (MD = −1.39 *P* < .0001) and for acute hemolysis (MD = −1.42%, *P* < .0001). For before and after studies, the use of VitE significantly improved the level of packed cell volume (MD = 0.56%, *P* < .00001), red blood cell half-life (MD = 2.19 days, *P* < .0001), and decreased the reticulocytes level (MD = −1.41%, *P* < .00001).

**Conclusion:**

Among patients with glucose-6-phosphate dehydrogenase deficiency, VitE might provide benefits such as increasing the hemoglobin, packed cell volume levels, red blood cell half-life, and decreasing the reticulocyte level, so reducing hemolysis. Further high-quality, well-designed randomized controlled trials are recommended.

## 1. Introduction

Glucose-6-phosphate dehydrogenase (G6PD) enzyme is a protein complex enzyme essential for red blood cells (RBCs) integrity and function.^[1]^ The G6PD enzyme is critical for protecting RBCs from oxidative stress.^[1,2]^ In the cellular metabolism within the pentose phosphate pathway, the G6PD enzyme is essential for the conversion of nicotinamide adenine dinucleotide phosphate (NADP) to NADP hydrogen (NADPH). The transformation of NADP to NADPH is necessary to form glutathione (GSH), a crucial antioxidant that protects erythrocytes from oxidative stress.^[3]^ G6PD deficiency is an x-linked disease that causes RBC hemolysis in response to oxidative stress.^[4,5]^ According to The National Organization for Rare Disorders, >400 million people live with G6PD deficiency.^[6]^ G6PD deficiency causes hemolysis in reaction to oxygen free radicals and reactive oxygen agents formed by stresses such as infections, specific foods and drugs, and even dietary or life habits.^[3,7,8]^ G6PD deficiency was grossly classified into 2 types; type A with mild deficiency and presented as a healthy person with recurrent episodes of hemolysis, and type B with marked deficiency and presented as chronic hemolytic anemia with recurrent attacks of hemolysis.^[1,9,10]^ The World Health Organization classified the G6PD deficiency variants into 5 classes according to the degree of enzyme deficiency and activity and hemolysis.^[11]^

Most patients with G6PD deficiency do not require therapy. Acute hemolytic anemia is mainly avoided in G6PD deficiency patients by avoiding fava beans, medicines, and substances that might produce oxidant stress. In order to effectively treat hemolysis in individuals with G6PD deficiency, it is essential to identify and discontinue the precipitating factor.^[4,8]^ To avoid renal damage caused by hemoglobin sedimentation, intravenous fluids may be administered with or without bicarbonate, and in certain situations, a blood transfusion may be required.^[9,12]^ In the most severe form of this condition, which is characterized by persistent hemolysis, treatment measures consist of splenectomy, blood transfusion, folic acid, and antioxidants.^[13]^

Evans and Bishop identified Vitamin E (VitE) (tocopherol) in 1922 as a dietary component essential for rat reproduction.^[14]^ It contributes to immunity and tissue regeneration. In humans, VitE insufficiency is linked to cystic fibrosis, ataxia, and abetalipoproteinemia (a condition that interferes with the proper absorption of fat and fat-soluble vitamins from meals), while in experimental animals, it is linked to frequent abortions and testicular degeneration. Additionally, VitE deficiency in preterm newborns may cause macrocytic anemia and increased hemolysis of RBCs. On the other hand, excessive VitE toxicity has been linked to headaches, weariness, dizziness, intestinal irritation, prolonged prothrombin time, and difficulty with the absorption of vitamins A and K.^[15–21]^ Due to the assumption that VitE, as an antioxidant, may reduce morbidity and mortality, VitE is currently one of the most regularly used dietary supplements.^[22]^ Based on this, it is thought that VitE may play a role in reducing hemolysis in patients with G6PD deficiency.^[23,24]^ As an antioxidant, VitE may benefit G6PD-deficient erythrocytes by protecting GSH from oxidation by free radical and peroxide-generating processes.^[25]^

We performed this systematic review and meta-analysis aiming to synthesize evidence from the published studies on the efficacy of VitE in treating and reducing hemolysis in patients with G6PD deficiency.

## 2. Methods

We followed the Preferred Reporting Items for Systematic Reviews and Meta-Analyses statement guidelines when reporting this systematic review and meta-analysis.^[26]^ All steps were done in strict accordance with the Cochrane Handbook of Systematic Reviews and Meta-analysis of Interventions.^[27]^ All steps of this study were prespecified, and the protocol was registered on PROSPERO (CRD42022333848).

### 2.1. Eligibility criteria

Studies were included in our review if they satisfied the following criteria:

Population: studies on patients with G6PD deficiency.

Intervention: studies where the experimental (or exposed) group received VitE.

Comparator: studies where the control group did not receive VitE or no control group.

Outcome: studies reported at least one of the following outcomes: serum hemoglobin, hematocrit, packed cell volume (PCV), RBC half-life, reticulocyte, blood GSH, and serum VitE levels.

Study design: comparative studies whose design was controlled trials with patients allocated to VitE or (placebo or no intervention in the control group) in a random or nonrandom allocation manner. We considered both double-blinded studies and open-label studies.

We also included prospective and retrospective observational studies and single-arm clinical trials with extreme caution; these studies were separated from randomized controlled trials (RCTs) in subgroups and were only considered in calculating the pooled effect size if their results were consistent with RCTs. In case of discrepancy between RCTs and observational studies, this was highlighted in the results, and the outcomes of RCTs were prioritized.

We excluded studies whose data were not reliable for extraction and analysis, studies reported as abstracts only or thesis, studies whose complete full texts were not available, and studies not published in the English language.

### 2.2. Information sources and search strategy

We performed a comprehensive search of 4 electronic databases (PubMed, Scopus, Web of Science, Cochrane Library, from inception until May 8, 2022, using Medical Subject Headings terms and using the following query: (Vitamin E OR Tocopherol OR Tocotrienol OR Tocovital OR VitaE OR Tocopharm) AND (GPD Deficiencies OR G6PD Deficiency OR Glucose 6 Phosphate Dehydrogenase Deficiency OR Glucosephosphate Dehydrogenase Deficiencies OR Glucose-6-Phosphate Dehydrogenase Deficiencies OR G6PD).

Further, the references of the included studies were manually searched for any potentially eligible studies. See Supplemental Digital Content S1, http://links.lww.com/MD/I466, which shows the detailed search strategy and results for each database.

### 2.3. Selection process

Duplicates were removed using Endnote (Clarivate Analytics, PA, USA), and the retrieved references were screened in 2 steps: the first step was to screen titles/abstracts of all identified articles independently by all authors to assess relevance to this meta-analysis, and the second step was to screen the full-text articles of the identified abstracts for final eligibility to meta-analysis.

### 2.4. Data collection process and data items

Data were extracted to a uniform data extraction sheet. The extracted data included characteristics of the included studies, characteristics of the population of included studies, risk of bias (ROB) domains, and outcome measures.

### 2.5. Assessing the ROB in the individual studies

We evaluated the quality of each included study by 2 authors independently. The Cochrane assessment tool was used for randomized clinical trials (ROB2).^[28]^ Risk Of Bias In Non-randomized Studies - of Interventions checklist was used for non-randomized studies of interventions. National Institutes of Health (NIH) tools were used for non-controlled before and after clinical trials^[29]^ and case series.^[30]^ A third author solved any disagreements.

### 2.6. Synthesis methods

The analysis was done using Review Manager Software (RevMan 5.4.1, The Nordic Cochrane Centre, The Cochrane Collaboration, Denmark) under the inverse variance method. As all outcomes were continuous, they were pooled as mean difference (MD) in a random effect model with a relative 95% confidence interval (CI). *P* value <.05 was considered statistically significant.

### 2.7. Choice of the meta-analysis model

We calculated the pooled effect size for all outcomes according to the DerSimonian Liard meta-analysis model. This random effect model assumes the included studies represent a random sample from the population and assigns a slightly higher weight to small studies on the expenses of larger studies. We chose this model because, unlike the fixed-effects model, it accommodates a larger standard error in the pooled estimate, making it suitable for inconsistent or controversial estimates. Thus, the calculated effects in our meta-analysis are conservative estimates that take into consideration the possible inconsistencies.

### 2.8. Assessment of heterogeneity

Heterogeneity among studies will be assessed by the chi-square test (Cochrane *Q* test) and using the *I*^2^ and *χ*^2^ tests. *χ*^2^ *P* value of <.1 will indicate significant heterogeneity. *I*^2^ values ≥50% were indicative of high heterogeneity.

### 2.9. Publication bias assessment

In the present study, we could not assess the existence of publication bias by Egger test for funnel plot asymmetry, as according to Egger and colleagues,^[31,32]^ publication bias assessment is unreliable for <10 pooled studies.

### 2.10. Certainty assessment

To test the robustness of the evidence, we conducted a certainty assessment through sensitivity analysis (also called leave-one-out meta-analysis). We ran sensitivity analysis in multiple scenarios for every outcome in the meta-analysis, excluding 1 study in each scenario to ensure the overall effect size was not dependent on any single study.

## 3. Results

### 3.1. Literature search results

**Figure 1.**
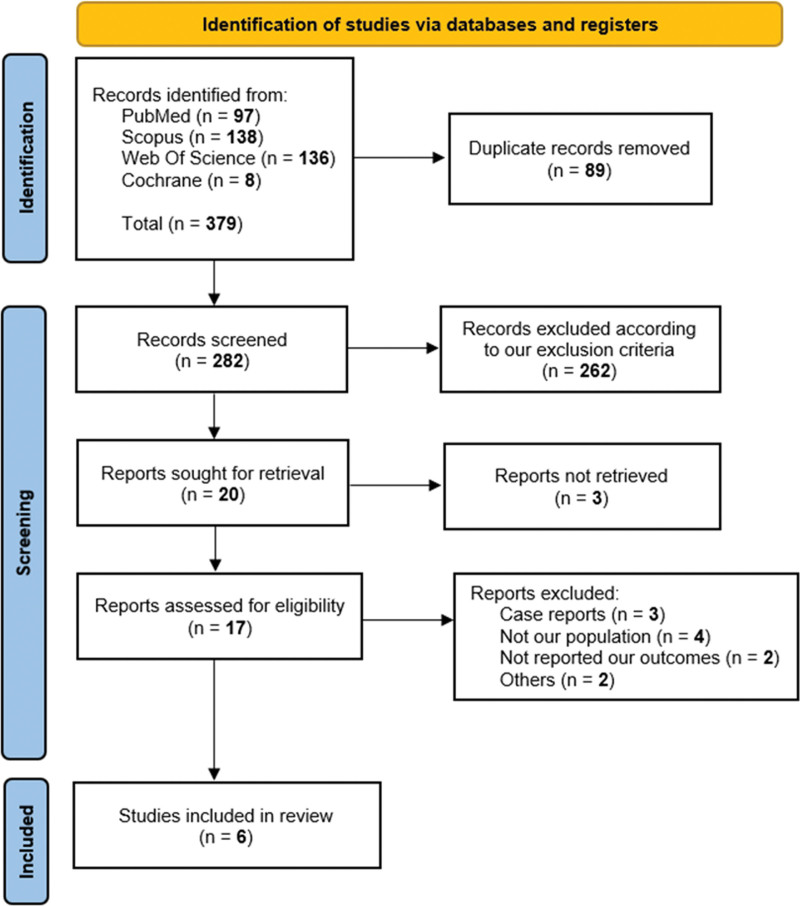
PRISMA flow diagram of studies’ screening and selection. PRISMA = Preferred Reporting Items for Systematic Reviews and Meta-Analyses.

Our literature search process retrieved 379 records. After duplicates were removed by the Endnote 20 (Clarivate Analytics, Philadelphia, PA), 282 records were screened. Following title and abstract screening, 20 articles were eligible for full-text screening. Of them, 6 studies were included in the meta-analysis.^[25,33–37]^ The references of the included studies were manually searched, and no further articles were included. The Preferred Reporting Items for Systematic Reviews and Meta-Analyses flow diagram of the study selection process is shown in Figure 1.

### 3.2. Characteristics of the included studies

Thirteen studies were included in the meta-analysis, with a total of 181 patients with hemolytic anemia due to G6PD deficiency. In 2 studies (128 patients), patients were assigned to receive either VitE or no VitE. In 4 studies (53 patients), the patients received VitE only with no control group. A summary of the characteristics of the included studies is provided in Tables 1 and 2. The included RCT had a high ROB according to the ROB2 tool, and the included non-randomized studies of interventions had a moderate ROB according to the Risk Of Bias In Non-randomized Studies - of Interventions checklists. According to the NIH tool, the 2 included pre-post clinical trials had good quality. One of the 2 included case series had a good quality, and the other had a fair quality according to the NIH tool. See Supplemental Digital Content S2, http://links.lww.com/MD/I467, which shows the details for each domain in the quality or ROB assessment for each study.

**Table 1:** Summary of the included studies.

Study ID	Title	Design	Country	Inclusion criteria	Exclusion criteria	Type of hemolysis	Groups	Dose	Treatment duration	Main results
Darbandi 2017^[35]^	The efficacy of Vitamin E and folic acid on the acute hemolysis caused by glucose-6 phosphate dehydrogenase	RCT	Iran	1–14 yr of age, with G6PDdef, hospitalized due to acute hemolysis, and no use of VitE or folic acid in the last mo.	Lack of testing follow-up, patient mortality before the completion of this study, and acute hemolysis caused by factors other than G6PD deficiency.	Acute	VitE	VitE daily (1–2 yr: 100 mg, 2–3 yr: 200 mg, 3–4 yr: 300 mg and >4 yr: 400 mg).	2 wk	↑ Hb levels ↓ reticulocytes combination with folic acid results in more improvement.
							Folic acid + VitE	Folic acid daily (1–2 yr: 2 mg and, >2 yr: 5mg). + vitamin E as group 1	2 wk	
							Control group	No supplement	2 wk	
Hafez 1986^[36]^	Improved erythrocyte survival with combined vitamin E and selenium therapy in children with glucose-6-phosphate dehydrogenase deficiency and mild chronic hemolysis	Non-controlled clinical trial (pre and post)	Egypt	Patients with chronic anemia with no clear cause, G6PD activity that was <1% of what is considered normal, no other health problems, and were not taking any medications. They hadn’t had acute hemolytic crisis or a transfusion in at least 2 mo.	Patients who were found to have ß-thalassemia trait because their red cells had a lower MCV and their A2 level was high were not included in the study.	Chronic	VitE	800 IU/d in divided doses, 4 times/ d	60 d	↑ Hb levels ↓ reticulocytes ↑ erythrocyte half-life the combination with selenium results in more improvement.
							VitE + selenium	VitE 800 IU/d + selenium 25 µg/d.	60 d	
Corash 1980 b^[33]^	Reduced chronic hemolysis during high-dose vitamin E administration in Mediterranean-type glucose-6-phosphate dehydrogenase deficiency	Non-controlled clinical trial (pre and post)	USA	Individuals of Greek citizenship living in the metropolitan region of Athens or in Patras, a small city in western Greece, and were referred due to G6PDdef diagnosis.	Beta-thalassemia trait patients, whose hemoglobin A2 level was raised and their MCV was reduced were not included.		VitE	800 IU	90 d and 1 yr in some patients	↑ Hb levels ↓ reticulocytes ↑ erythrocyte half-life
Corash 1982 (3 kindreds)^[25]^	No title (part of other paper)	Case series (pre and post)	USA	G6PDdef kindreds with chronic hemolysis	NR	Chronic	VitE	1000 IU/d in divided doses	6 mo	No improvement
Johnson 1983^[37]^	High-dose vitamin E does not decrease the rate of chronic hemolysis in glucose-6-phosphate dehydrogenase deficiency	Case series (pre and post)	USA	3 men with G6PD-deficiency.	NR	Chronic	VitE	2000–2400 IU/d (30 IU/kg/d)	4 wk	No improvement
Sultana 2006^[34]^	Effects of vitamin E supplementation on some aspects of hematological variables in patients of hemolytic anemia with glucose 6 phosphate dehydrogenase (G6PD) deficiency	Non-randomized controlled trial	Bangladesh	Subjects with age (5–40) yr with G6PDdef were included.	Patients with ß-thalassemia trait and patients with acute hemolytic crisis or who had recently undergone blood transfusions were excluded from this study.	Chronic	VitE	800 IU/d for adult and 400 IU/d for children ≤12 yr	60 d	↑ Hb levels ↓ reticulocytes ↑ PCV ↑ erythrocyte number ↓ serum bilirubin
							Control group	No supplement	60 d	

G6PDdef = glucose-6-phosphate dehydrogenase enzyme deficiency, Hb = hemoglobin, PCV = packed cell volume, RCT = randomized controlled trial, VitE = vitamin E.

**Table 2:** Baseline characteristics of the included studies.

Study ID	Groups	No. of participants	Age		Sex (males)		Hemoglobin (g/dL)		RBC half-life (d)		Reticulocyte (%)		Serum vitamin E (mg/dL)	
			Mean	SD	Number	%	Mean	SD	Mean	SD	Mean	SD	Mean	SD
Darbandi 2017^[35]^	VitE	30	36.93	NR	21	70	6.93	1.41	NR		3.41	1.56	NR	
	Folic acid + VitE	30	44.63	NR	19	63.7	6.62	1.56			3.03	1.17		
	Control group	30	43.73	NR	18	60	6.68	1.6	NR		2.76	1.4	NR	
Hafez 1986^[36]^	VitE	18	3–12		18	100	9	1.5	16.9	3.05	3	0.68	0.5	0.025
	VitE + selenium	18			18	100	8.6	1.74	15.6	8.2	2.8	0.55	0.53	0.13
Corash 1980 b^[33]^	VitE	23	15	9.75	20	87	13.3	0.959	22.9	3.13	2.8	0.959	0.53	0.169
Corash 1982 (3 kindreds)^[25]^	VitE	9	NR		7	77.78	12.7	0.933	9.3	6.3	12.2	4.3	0.87	0.24
Johnson 1983^[37]^	VitE	3	32.67	16.17	3	100%	13.3	0.72	6.9	1	24	18.2	1.05	0.32
Sultana 2006^[34]^	VitE	34	5–40		NR		10.34	1	NR		2.79	0.55	NR	
	Control group	34					10.15	0.94			3.06	0.44		

NR = not reported, RBC = red blood cell, SD = standard deviation, VitE = vitamin E.

### 3.3. Analysis of RCTs

#### 1.3.3. Hemoglobin (g/dL).

**Figure 2.**
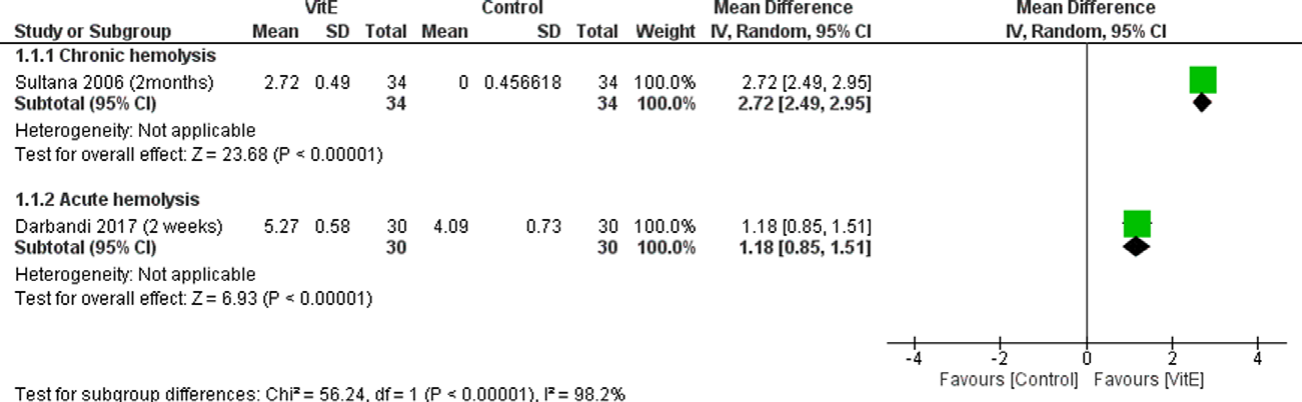
Forest plot comparing VitE and control groups regarding hemoglobin level. VitE = vitamin E.

The overall MD between the VitE and the control groups in rising serum hemoglobin levels favors the VitE group in both acute and chronic hemolysis, Figure 2. For chronic hemolysis, (MD = 2.72 g/dL, 95% CI [2.49–2.95], *P* < .0001). For acute hemolysis, (MD = 1.18 g/dL, 95% CI [0.85–1.51], *P* < .0001).

#### 2.3.3. Reticulocytes (%).

**Figure 3.**
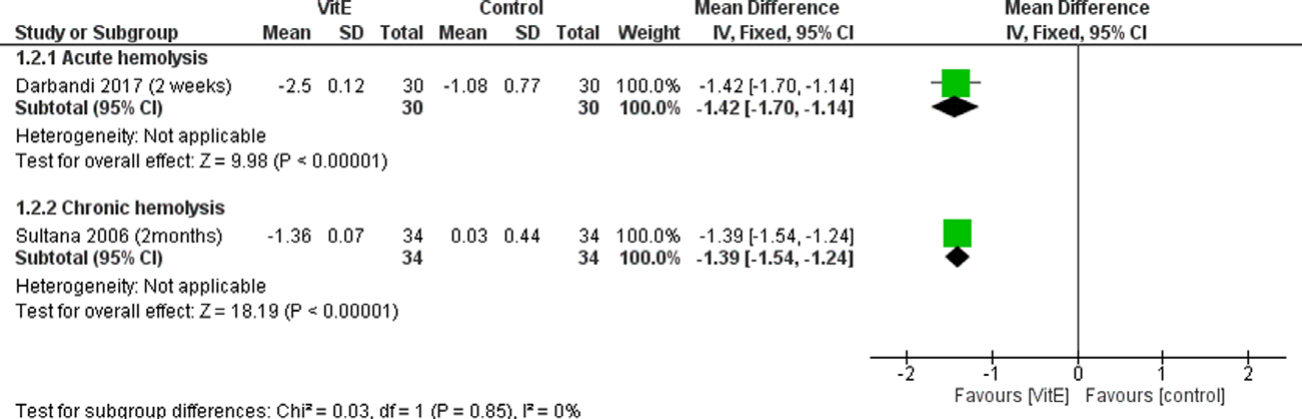
Forest plot comparing VitE and control groups regarding the reticulocytes. VitE = vitamin E.

The overall MD between the VitE group and the control group in decreasing the reticulocyte levels favors the VitE group in both, Figure 3. For chronic hemolysis, (MD = −1.39%, 95% CI [−1.54–−1.24], *P* < .0001). For acute hemolysis, (MD = −1.42%, 95% CI [−1.70–−1.14], *P* < .0001).

### 3.4. Analysis of pre- and post-studies (all were chronic hemolysis)

#### 1.3.4. Hemoglobin (g/dL).

The overall mean change from baseline after VitE supplementation showed no significant increase in the hemoglobin level, (MD = 0.99 g/dL, 95% CI [−0.02–2.00], *P* = .05). There was disagreement between the results from RCT and single-arm pre and post trials. The results from RCT showed an increase in the hemoglobin level (MD = 3.98 g/dL, 95% CI [1.48–6.47], *P* = .002). However, pre- and post-studies showed no significant increase in the hemoglobin level (MD = 0.46 g/dL, 95% CI [−0.07–1.00], *P* = .09); see Supplemental Figure S1, Supplemental Digital Content, http://links.lww.com/MD/I468.

#### 2.3.4. PCV (%).

The overall mean change from baseline after VitE supplementation showed an increase in the PCV level, (MD = 0.56%, 95% CI [0.42–0.71], *P* < .00001). We divided the data into 2 subgroups following our protocol. For the RCT subgroup, (MD = 0.56%, 95% CI [0.41–0.71], *P* < .00001). For pre and post subgroup, (MD = 2.10%, 95% CI [−0.62–4.82], *P* = .13). Pooled studies were homogenous in the pre and post subgroup (*χ*^2^ *P* = .76, *I*^2^ = 0%), and the heterogeneity was not applicable to the RCT subgroup; see Supplemental Figure S2, Supplemental Digital Content, http://links.lww.com/MD/I469.

#### 3.3.4. RBC half-life (days).

The overall mean change from baseline after VitE supplementation showed an increase in the RBCs half-life (MD = 2.19 day, 95% CI [1.32–3.07], *P* < .0001). Pooled studies were homogenous (*χ*^2^ *P* = .98, *I*^2^ = 0%); see Supplemental Figure S3, Supplemental Digital Content, http://links.lww.com/MD/I470.

#### 4.3.4. Reticulocyte (%).

The overall mean change from baseline after VitE supplementation showed a decrease in the reticulocyte level (MD = −1.41%, 95% CI [−2.03–−0.79], *P* < .00001). We divided the data into 2 subgroups following our protocol. For the RCT subgroup, (MD = −1.39%, 95% CI [−1.54–−1.24], *P* < .0001). For pre and post subgroup, (MD = −1.26%, 95% CI [−2.47–−0.06], *P* = .04). In the RCT subgroup, the heterogeneity was not applicable. In the pre- and post-subgroups, pooled studies were not homogenous (*χ*^2^ *P* = .004, *I*^2^ = 82%); see Supplemental Figure S4, Supplemental Digital Content, http://links.lww.com/MD/I471.

#### 5.3.4. Serum VitE (mg/dL).

The overall mean change from baseline after VitE supplementation showed an increase in the serum VitE level, (MD = 1.04 mg/dL, 95% CI [0.13–1.94], *P* = .03). Pooled studies were homogenous (*χ*^2^ *P* = .95, *I*^2^ = 0%); see Supplemental Figure S5, Supplemental Digital Content, http://links.lww.com/MD/I472.

## 4. Discussion

### 4.1. Significance of the study

To the best of our knowledge, this is the first meta-analysis studying the role of VitE in G6PD deficiency patients. Despite the advanced in research, current treatment options for patients with G6PD deficiency are still limited. This work expands the literature by providing evidence on the effects of VitE supplementation in patients with G6PD deficiency. The significance of this paper is not only to summarize the evidence of the use of VitE in G6PD deficiency patients but also to evaluate the theory and open the door in front of future research about the use of any antioxidants in patients with G6PD deficiency.

### 4.2. Summary of findings

Data from the single arm pre- and post-trials showed that among patients with G6PD deficiency, VitE supplementations significantly increased PCV, and RBCs half-life, VitE level, and decreased the reticulocyte level. While evidence from controlled studies showed that VitE significantly increased the hemoglobin level in patients with both chronic and acute hemolysis compared to the standard of care. VitE also significantly decrease the reticulocyte level in comparison with the control group.

### 4.3. Introduction to pathogenesis

G6PD deficiency grossly has 2 types; type A with mild deficiency and presented as a healthy person with recurrent episodes of hemolysis, and type B with marked deficiency and presented as chronic hemolytic anemia with recurrent attacks of hemolysis.^[38]^ In our review, most of the included studies in the analysis investigated the efficacy of VitE on patients with chronic hemolysis^[25,33,34,36,37]^ and only 1 study investigated the efficacy of acute hemolysis.^[35]^

The formation of NADPH from the NADP^+^ in the pentose phosphate pathway protects the cells from oxidative stress, as it helps in the regeneration of the reduced GSH. The sulfhydryl groups of hemoglobin that are sensitive to oxidation by oxygen radicals and hydrogen peroxide are shielded by this GSH.^[2,9]^ The formation of NADPH requires the G6PD enzyme. In patients with G6PD deficiency, there is a decrease in the formation of NADPH according to the degree of deficiency and the degree of the activity of the enzyme, by which the World Health Organization classify the disease.^[11]^ The most affected cells by this deficiency are the RBCs, although the deficiency is present in all cells because the only source of NADPH in RBCs is pentose phosphate pathway.^[9]^ VitE protects the RBCs cell membrane from oxidative stress as an antioxidant.^[23,25,39]^

### 4.4. Explanation of the findings

**Figure 4.**
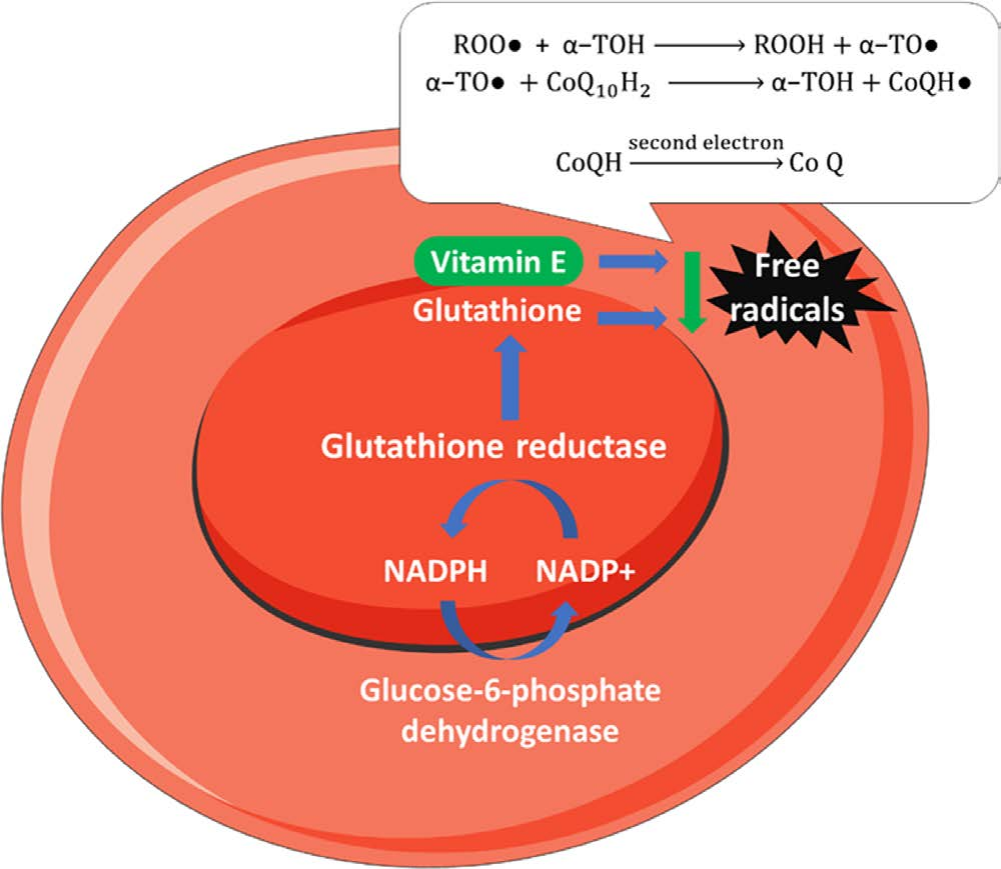
How VitE protects the RBCs membrane. RBCs = red blood cells, VitE = vitamin E.

Some studies reported that serum VitE was below the normal level in patients with G6PD deficiency^[39–41]^ and were notified that low VitE level was associated with low RBC survival and increased its susceptibility to oxidative stress.^[42,43]^ VitE supplementation in preterm newborns with hyperbilirubinemia resulted in a considerable decrease in serum bilirubin level, which is thought to be produced by a reduction in RBCs hemolysis.^[44]^ The underlying mechanism of its effect is that VitE captures and neutralizes the already present free radicals and limits the creation of new free radicals by different mechanisms, Figure 4. Thus, VitE may aid in preventing or delaying chronic illnesses related to reactive oxygen species molecules, as in G6PD deficiency.^[45–47]^ So, VitE may have a protective role in the long-term complications of chronic hemolysis such as pigment gallstone and renal dysfunction with chronic hemoglobin sedimentation in the kidney.^[48]^

### 4.5. Explanation of the heterogeneity

In reticulocyte level, the heterogeneity contributed to that (Corash 1982 [3 kindreds]) measured the reticulocyte level at 6 months, a longer time than other studies, so a more clear effect was observed.

### 4.6. Agreement and disagreement with previous studies

In agreement with our results, Koriem et al^[49]^ a study published in 2017, showed that G6PD deficiency patients with favism who take VitE for one month had a high level of hemoglobin, hematocrit, and serum G6PD, which was dose-dependent, increasing the dose of VitE was associated with better outcomes. We cannot include this study in our analysis as it reported the results without reporting the baseline values, so we cannot calculate the mean change. In contrast, Johnson et al^[37]^ reported a case series of three G6PD deficiency patients, and they showed no reduction in chronic hemolysis after VitE supplementation. Newman et al^[23]^ also reported a case of a G6PD deficiency patient who showed no improvement after VitE supplementation, although it protects the membrane of RBCs from lipid peroxidation. The explanation reported by this paper was that this protection was not enough to improve the clinical status of the patient.

### 4.7. Strength points and limitations

To the best of our knowledge, this is the first meta-analysis to evaluate the efficacy of VitE in patients with G6PD deficiency. The study has a few limitations: the small number of included studies in general, and specifically, no comparison between the different doses of VitE; different study designs; only 2 RCTs investigated the efficacy of VitE in G6PD deficiency patients; and low certainty in the overall evidence. Therefore, future RCTs are required to confirm these findings.

### 4.8. Recommendations for future research and clinical practice

Although the number of studies was small, we could generate synthesis which can direct further research towards making well-designed, high-quality, RCTs investigating the different doses of VitE, as our results showed a promising beneficial effect of VitE in G6PD deficiency patients, especially with chronic hemolysis. Conducting a RCT on neonates with G6PD deficiency would be valuable as well. We also recommend investigating the actual cost of utilizing VitE in long-term supplementation. Due to the safety of VitE with low rate and non-severe side effects,^[50]^ until the development of stronger evidence, we recommend using VitE in patients with G6PD deficiency as a supplementary with the standard management and as a long-term preventive supplementation in patients with chronic hemolysis.

## 5. Conclusion

Among patients with G6PD deficiency, VitE might provide benefits such as increasing the hemoglobin, PCV levels, RBCs half-life and decreasing the reticulocyte level, so reducing hemolysis. Although there is a low level of evidence, VitE can be used until higher-quality evidence is developed, as it seems safe without side effects. Further high-quality, well-designed RCTs are recommended to confirm or deny the current evidence.

## Author contributions

**Conceptualization:** Mahmoud Allam.

**Data curation:** Ali Seif, Mahmoud Allam.

**Formal analysis:** Omar Ahmed Abdelwahab.

**Investigation:** Khaled Akil, Ali Seif.

**Methodology:** Omar Ahmed Abdelwahab, Khaled Akil, Ali Seif.

**Resources:** Khaled Akil.

**Software:** Omar Ahmed Abdelwahab.

**Supervision:** Mohamed El-Sebaey Sherif, Mohamed N. Al-Alfy.

**Validation:** Omar Ahmed Abdelwahab, Mohamed N. Al-Alfy.

**Visualization:** Mohamed N. Al-Alfy.

**Writing – original draft:** Omar Ahmed Abdelwahab, Mahmoud Allam, Mohamed El-Sebaey Sherif.

## Supplementary Material














