# Supplementary material for: The potential role of vitamin E in patients with glucose-6-phosphate dehydrogenase deficiency: A systematic review and meta-analysis
Source: Medicine (Baltimore). 2023 Feb 10;102(6):e32937. doi: 10.1097/MD.0000000000032937 (PMC9907971; doi:10.1097/MD.0000000000032937)
Supplement: Supplementary file 1 [file medi-102-e32937-s001.pdf]

1. Search strategy for each database and the results of this search.

Pubmed

Strategy: (Vitamin E OR Tocopherol OR Tocotrienol OR Tocovital OR VitaE OR Tocopharm) AND (GPD Deficiencies OR G6PD Deficiency OR Glucose 6 Phosphate Dehydrogenase Deficiency OR Glucosephosphate Dehydrogenase Deficiencies OR Glucose-6-Phosphate Dehydrogenase Deficiencies OR G6PD)

Results: 97

Scopus

Strategy: TITLE-ABS-KEY (("Vitamin E" OR tocopherol OR tocotrienol OR tocovital OR vitae OR tocopharm) AND (favism OR "GPD Deficiencies" OR "G6PD Deficiency" OR "Glucose 6 Phosphate Dehydrogenase Deficiency" OR "Glucosephosphate Dehydrogenase Deficiencies" OR "GPD Deficiency" OR "Glucose-6-Phosphate Dehydrogenase Deficiencies" OR g6pd))

Results: 138

Cochrane

Strategy: (Vitamin E OR Tocopherol OR Tocotrienol OR Tocovital OR VitaE OR Tocopharm) AND (GPD Deficiencies OR G6PD Deficiency OR Glucose 6 Phosphate Dehydrogenase Deficiency OR Glucosephosphate Dehydrogenase Deficiencies OR Glucose-6-Phosphate Dehydrogenase Deficiencies OR G6PD)

Results: 8

WOS

Strategy: (Vitamin E OR Tocopherol OR Tocotrienol OR Tocovital OR VitaE OR Tocopharm) AND (GPD Deficiencies OR G6PD Deficiency OR Glucose 6 Phosphate Dehydrogenase Deficiency OR Glucosephosphate Dehydrogenase Deficiencies OR Glucose-6-Phosphate Dehydrogenase Deficiencies OR G6PD)

Results: 136
