# Supplementary material for: The potential role of vitamin E in patients with glucose-6-phosphate dehydrogenase deficiency: A systematic review and meta-analysis
Source: Medicine (Baltimore). 2023 Feb 10;102(6):e32937. doi: 10.1097/MD.0000000000032937 (PMC9907971; doi:10.1097/MD.0000000000032937)
Supplement: Supplementary file 2 [file medi-102-e32937-s002.pdf]

1. Risk of bias and quality assessment for each study

Study ID	ROB assessment for RCT according to ROB2 tool												
Darbandi 2017	D1	D2	D3	D4	D5	Other bias							**Overall bias**
	SC	HR	LR	LR	SC	Low risk							High risk of bias
Study ID	ROB assessment for NRSI according to ROBINS1 tool												
Sultana 2006	D1	D2	D3	D4	D5	D6	D7						**Overall bias**
	LR	LR	LR	MR	LR	LR	LR						Moderate risk of bias
Study ID	Quality assessment for before-after (Pre-Post) Studies with no control group according to NIH tool												
	Q1	Q2	Q3	Q4	Q5	Q6	Q7	Q8	Q9	Q10	Q11	Q12	**Overall quality**
Hafez 1986	Y	Y	Y	Y	CD	Y	Y	N	Y	Y	CD	NA	Good (8)
Corash 1980 b	Y	Y	Y	Y	N	Y	Y	N	Y	Y	CD	NA	Good (8)
Study ID	Quality assessment for before-after (Pre-Post) Studies with no control group according to NIH tool												
	Q1	Q2	Q3	Q4	Q5	Q6	Q7	Q8	Q9				**Overall quality**
Corash 1982 (3 kindreds)	Y	Y	Y	N	Y	Y	Y	Y	Y				Good (8)
Johnson 1983	Y	Y	N	N	Y	Y	N	N	Y				Fair (5)

ROB2 tool: bias due to/ in: D1: randomization process; D2: deviations from intended interventions; D3: measurement of the outcome; D4: missing outcome data; D5: selection of the reported result. **ROBINS1 tool**: bias due to/ in: D1: confounding; D2: selection of participants into the study; D3: classification of

interventions; D4: deviations from intended interventions; D5: missing data; D6: measurement of outcomes; D7: selection of the reported result. **NIH tool for pre and post studies**: Q1: Objective clearly stated; Q2: eligibility criteria described; Q3: representative patient population; Q4: all eligible participants enrolled in study; Q5: sufficient sample size; Q6: intervention described; Q7: outcome measures specified; Q8: outcome assessors blinded; Q9: loss to follow-up; Q10: statistical analysis of outcome measures before and after intervention; Q11: interrupted time-series design; Q12: individual data used for group-level effects. **NIH tool for case series**: Q1: Was study question or objective clearly stated?, Q2: Was study population clearly and fully described, including case definition?, Q3: Were cases consecutive?, Q4: Were subjects comparable?, Q5: Was intervention clearly described?, Q6: Were outcome measures clearly defined, valid, reliable, and implemented consistently across all study participants?, Q7: Was length of follow-up adequate?, Q8: Were statistical methods well-described?, Q9: Were results well-described?.

LR: low risk; MR: moderate risk; HR, high risk; SC: some concerns; Y, yes; N, no; NA, not applicable; CD: cannot determined.
