# Supplementary material for: The potential role of vitamin E in patients with glucose-6-phosphate dehydrogenase deficiency: A systematic review and meta-analysis
Source: Medicine (Baltimore). 2023 Feb 10;102(6):e32937. doi: 10.1097/MD.0000000000032937 (PMC9907971; doi:10.1097/MD.0000000000032937)
Supplement: Supplementary file 3 [file medi-102-e32937-s003.pdf]

Supplemental Figure S1: *Forest plot comparing the change from baseline after VitE regarding hemoglobin level.*

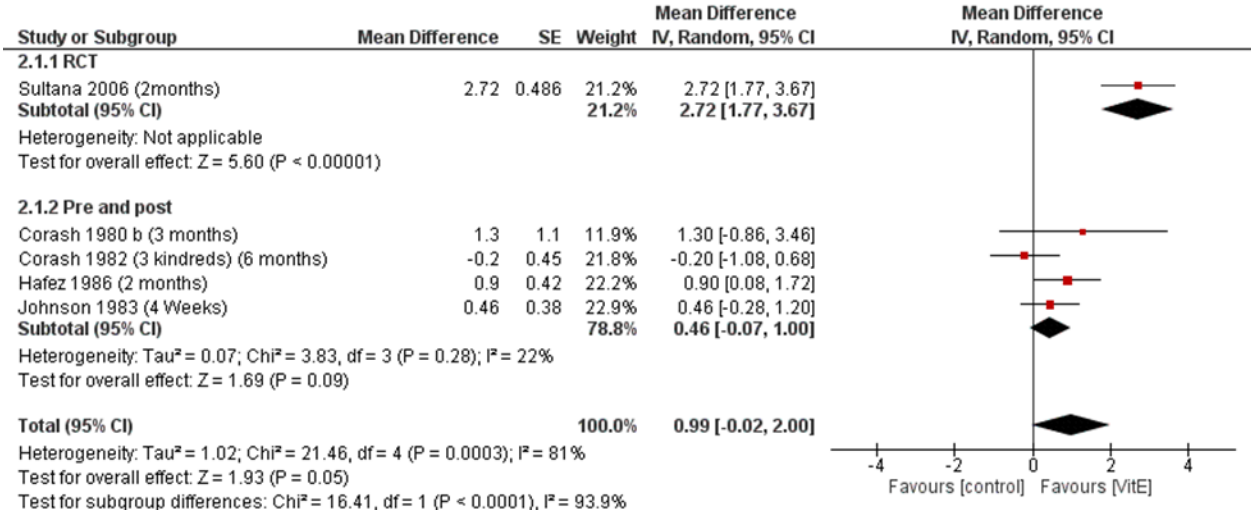

Study or Subgroup	Mean Difference	SE	Weight	Mean Difference IV, Random, 95% CI
**2.1.1 RCT**				
Sultana 2006 (2months)	2.72	0.486	21.2%	2.72 [1.77, 3.67]
**Subtotal (95% CI)**			**21.2%**	**2.72 [1.77, 3.67]**
Heterogeneity: Not applicable				
Test for overall effect: Z = 5.60 (P < 0.00001)				
**2.1.2 Pre and post**				
Corash 1980 b (3 months)	1.3	1.1	11.9%	1.30 [-0.86, 3.46]
Corash 1982 (3 kindreds) (6 months)	-0.2	0.45	21.8%	-0.20 [-1.08, 0.68]
Hafez 1986 (2 months)	0.9	0.42	22.2%	0.90 [0.08, 1.72]
Johnson 1983 (4 Weeks)	0.46	0.38	22.9%	0.46 [-0.28, 1.20]
**Subtotal (95% CI)**			**78.8%**	**0.46 [-0.07, 1.00]**
Heterogeneity: $Tau^2 = 0.07$; $Chi^2 = 3.83$, df = 3 (P = 0.28); $I^2 = 22\%$				
Test for overall effect: Z = 1.69 (P = 0.09)				
**Total (95% CI)**			**100.0%**	**0.99 [-0.02, 2.00]**
Heterogeneity: $Tau^2 = 1.02$; $Chi^2 = 21.46$, df = 4 (P = 0.0003); $I^2 = 81\%$				
Test for overall effect: Z = 1.93 (P = 0.05)				
Test for subgroup differences: $Chi^2 = 16.41$, df = 1 (P < 0.0001), $I^2 = 93.9\%$				
