# Supplementary material for: The potential role of vitamin E in patients with glucose-6-phosphate dehydrogenase deficiency: A systematic review and meta-analysis
Source: Medicine (Baltimore). 2023 Feb 10;102(6):e32937. doi: 10.1097/MD.0000000000032937 (PMC9907971; doi:10.1097/MD.0000000000032937)

Supplemental Figure S2: *Forest plot comparing the change from baseline after VitE regarding PCV level.*

Study or Subgroup	Mean Difference	SE	Weight	Mean Difference IV, Random, 95% CI
**2.3.2 RCT**				
Sultana 2006 (2months)	0.56	0.076	99.7%	0.56 [0.41, 0.71]
**Subtotal (95% CI)**			**99.7%**	**0.56 [0.41, 0.71]**
Heterogeneity: Not applicable				
Test for overall effect: Z = 7.37 (P < 0.00001)				
**2.3.3 Pre and post**				
Corash 1980 b (3 months)	1.5	2.4	0.1%	1.50 [-3.20, 6.20]
Hafez 1986 (2 months)	2.4	1.7	0.2%	2.40 [-0.93, 5.73]
**Subtotal (95% CI)**			**0.3%**	**2.10 [-0.62, 4.82]**
Heterogeneity: Tau² = 0.00; Chi² = 0.09, df = 1 (P = 0.76); I² = 0%				
Test for overall effect: Z = 1.51 (P = 0.13)				
**Total (95% CI)**			**100.0%**	**0.56 [0.42, 0.71]**
Heterogeneity: Tau² = 0.00; Chi² = 1.32, df = 2 (P = 0.52); I² = 0%				
Test for overall effect: Z = 7.44 (P < 0.00001)				
Test for subgroup differences: Chi² = 1.23, df = 1 (P = 0.27), I² = 18.5%				

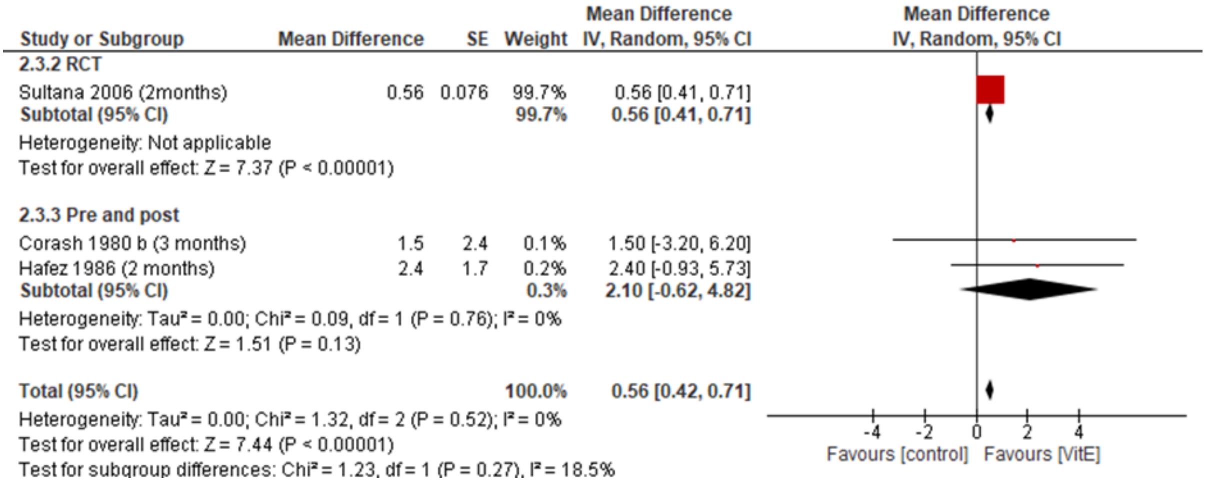

Supplement: Supplementary file 4 [file medi-102-e32937-s004.pdf]
