# Supplementary material for: The potential role of vitamin E in patients with glucose-6-phosphate dehydrogenase deficiency: A systematic review and meta-analysis
Source: Medicine (Baltimore). 2023 Feb 10;102(6):e32937. doi: 10.1097/MD.0000000000032937 (PMC9907971; doi:10.1097/MD.0000000000032937)

Supplemental Figure S3: *Forest plot comparing the change from baseline after VitE regarding RBCs half-life.*

Study or Subgroup	Mean Difference	SE	Weight	Mean Difference IV, Fixed, 95% CI
Corash 1980 b (3 months)	2.2	0.45	99.4%	2.20 [1.32, 3.08]
Corash 1982 (3 kindreds) (6 months)	0.6	9.7	0.2%	0.60 [-18.41, 19.61]
Hafez 1986 (2 months)	5.9	12.73	0.1%	5.90 [-19.05, 30.85]
Johnson 1983 (4 Weeks)	-0.334	8.83	0.3%	-0.33 [-17.64, 16.97]
**Total (95% CI)**			**100.0%**	**2.19 [1.32, 3.07]**

Heterogeneity: Chi² = 0.19, df = 3 (P = 0.98); I² = 0%
Test for overall effect: Z = 4.89 (P < 0.00001)

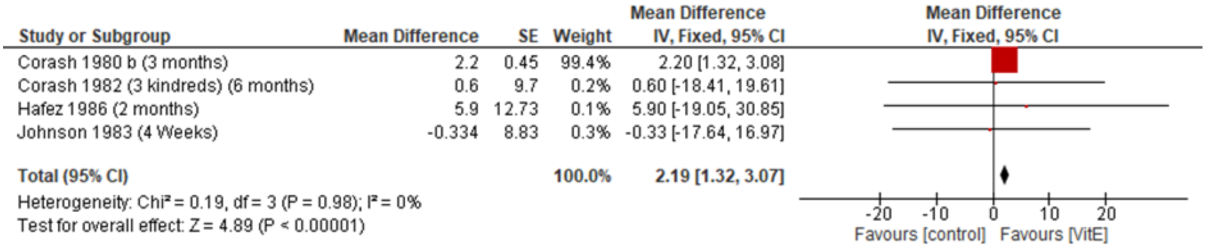

Supplement: Supplementary file 5 [file medi-102-e32937-s005.pdf]
