# Supplementary material for: The potential role of vitamin E in patients with glucose-6-phosphate dehydrogenase deficiency: A systematic review and meta-analysis
Source: Medicine (Baltimore). 2023 Feb 10;102(6):e32937. doi: 10.1097/MD.0000000000032937 (PMC9907971; doi:10.1097/MD.0000000000032937)
Supplement: Supplementary file 6 [file medi-102-e32937-s006.pdf]

Supplemental Figure S4: *Forest plot comparing the change from baseline after VitE regarding the reticulocytes level.*

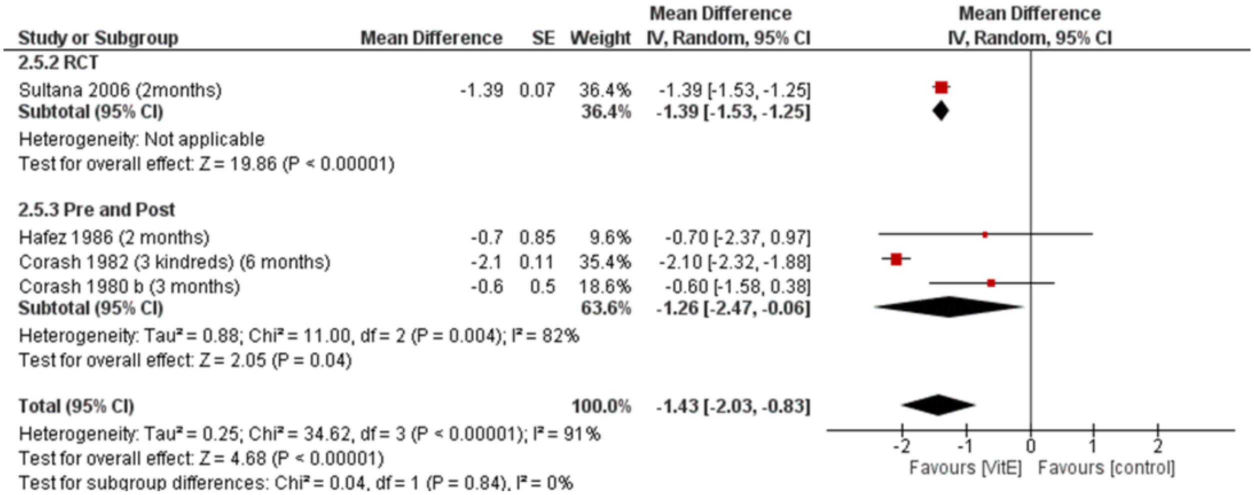

Study or Subgroup	Mean Difference	SE	Weight	Mean Difference IV, Random, 95% CI
**2.5.2 RCT**				
Sultana 2006 (2months)	-1.39	0.07	36.4%	-1.39 [-1.53, -1.25]
**Subtotal (95% CI)**			**36.4%**	**-1.39 [-1.53, -1.25]**
Heterogeneity: Not applicable				
Test for overall effect: Z = 19.86 (P < 0.00001)				
**2.5.3 Pre and Post**				
Hafez 1986 (2 months)	-0.7	0.85	9.6%	-0.70 [-2.37, 0.97]
Corash 1982 (3 kindreds) (6 months)	-2.1	0.11	35.4%	-2.10 [-2.32, -1.88]
Corash 1980 b (3 months)	-0.6	0.5	18.6%	-0.60 [-1.58, 0.38]
**Subtotal (95% CI)**			**63.6%**	**-1.26 [-2.47, -0.06]**
Heterogeneity: Tau² = 0.88; Chi² = 11.00, df = 2 (P = 0.004); I² = 82%				
Test for overall effect: Z = 2.05 (P = 0.04)				
**Total (95% CI)**			**100.0%**	**-1.43 [-2.03, -0.83]**
Heterogeneity: Tau² = 0.25; Chi² = 34.62, df = 3 (P < 0.00001); I² = 91%				
Test for overall effect: Z = 4.68 (P < 0.00001)				
Test for subgroup differences: Chi² = 0.04, df = 1 (P = 0.84), I² = 0%				
