# Supplementary material for: The potential role of vitamin E in patients with glucose-6-phosphate dehydrogenase deficiency: A systematic review and meta-analysis
Source: Medicine (Baltimore). 2023 Feb 10;102(6):e32937. doi: 10.1097/MD.0000000000032937 (PMC9907971; doi:10.1097/MD.0000000000032937)
Supplement: Supplementary file 7 [file medi-102-e32937-s007.pdf]

Supplemental Figure S5: *Forest plot comparing the change from baseline after VitE regarding serum VitE.*

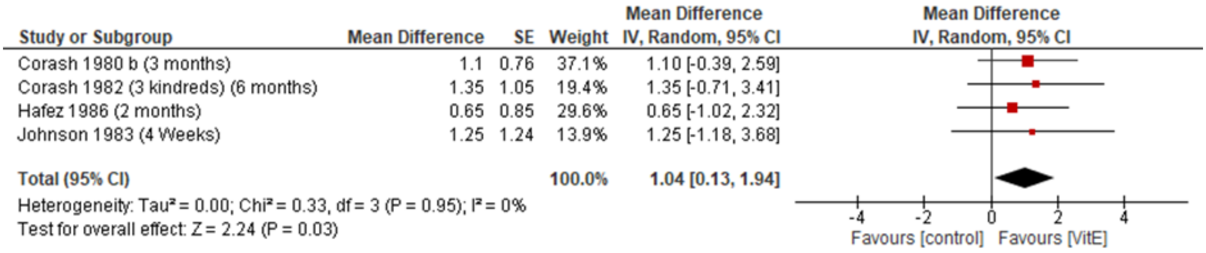

Study or Subgroup	Mean Difference	SE	Weight	Mean Difference IV, Random, 95% CI
Corash 1980 b (3 months)	1.1	0.76	37.1%	1.10 [-0.39, 2.59]
Corash 1982 (3 kindreds) (6 months)	1.35	1.05	19.4%	1.35 [-0.71, 3.41]
Hafez 1986 (2 months)	0.65	0.85	29.6%	0.65 [-1.02, 2.32]
Johnson 1983 (4 Weeks)	1.25	1.24	13.9%	1.25 [-1.18, 3.68]
**Total (95% CI)**			**100.0%**	**1.04 [0.13, 1.94]**

Heterogeneity: Tau² = 0.00; Chi² = 0.33, df = 3 (P = 0.95); I² = 0%

Test for overall effect: Z = 2.24 (P = 0.03)
